# Manual cell selection in single cell transcriptomics using scSELpy supports the analysis of immune cell subsets

**DOI:** 10.3389/fimmu.2023.1027346

**Published:** 2023-04-25

**Authors:** Mark Dedden, Maximilian Wiendl, Tanja M. Müller, Markus F. Neurath, Sebastian Zundler

**Affiliations:** ^1^ Department of Medicine 1, University Hospital Erlangen and Friedrich-Alexander-Universität Erlangen-Nürnberg, Erlangen, Germany; ^2^ Deutsches Zentrum Immuntherapie (DZI), University Hospital Erlangen, Erlangen, Germany

**Keywords:** single cell RNA sequencing, transcriptomics, chronic inflammation, inflammatory bowel disease, gut homing

## Abstract

**Introduction:**

Single cell RNA sequencing plays an increasing and indispensable role in immunological research such as in the field of inflammatory bowel diseases (IBD). Professional pipelines are complex, but tools for the manual selection and further downstream analysis of single cell populations are missing so far.

**Methods:**

We developed a tool called scSELpy, which can easily be integrated into Scanpy-based pipelines, allowing the manual selection of cells on single cell transcriptomic datasets by drawing polygons on various data representations. The tool further supports the downstream analysis of the selected cells and the plotting of results.

**Results:**

Taking advantage of two previously published single cell RNA sequencing datasets we show that this tool is useful for the positive and negative selection of T cell subsets implicated in IBD beyond standard clustering. We further demonstrate the feasibility for subphenotyping T cell subsets and use scSELpy to corroborate earlier conclusions drawn from the dataset. Moreover, we also show its usefulness in the context of T cell receptor sequencing.

**Discussion:**

Collectively, scSELpy is a promising additive tool fulfilling a so far unmet need in the field of single cell transcriptomic analysis that might support future immunological research.

## Introduction

Progress in life sciences has led to deep insights into physiological and pathological processes in recent years. However, in turn, this also resulted in more and more sophisticated problems to be further addressed. In particular, this concerns the inconceivable complexity of immunological processes that research has discovered and now tries to understand in even greater detail.

One example are immune-mediated inflammatory disorders (IMIDs) (1) such as the inflammatory bowel diseases (IBD) ulcerative colitis (UC) and Crohn’s disease (CD) characterized by relapsing-remitting chronic inflammation of the gastrointestinal tract (2, 3). While an important role of the immune system in the pathogenesis of IBD had early been demonstrated (4), further ground-breaking insights in fields such as genetics (5) have shaped the current picture of multifactorial diseases driven by antigen translocation from a dysbiotic luminal microenvironment over a leaky epithelial barrier into the lamina propria of a genetically susceptible individual (6). Here, dysregulated immune responses are evoked and cause inflammation resulting in tissue destruction and a vicious cycle of further host-environment miscommunication (7). This also triggers the recruitment of immune cells such as T lymphocytes from the peripheral blood to the intestinal tissue, a process called gut homing that is specifically regulated in the gut and involves molecules such as the chemokine receptor CX3CR1 or the integrin α4β7 (8). This also contributes to the expansion of diverse pro-inflammatory effector and effector memory T cell subsets in the inflamed gut including T helper 1 (TH1) and T helper 17 (TH17) cells (9), while regulatory T cells cannot sufficiently suppress these disease-driving cells (10).

Technological advances and also the introduction of novel therapeutic approaches (11–13) into the clinics have led to unprecedented insights into the regulation of aberrant immune responses in chronic inflammation in general and IBD in particular. In this context, the single cell RNA-sequencing (scRNA-seq) technology has become a popular and indispensable technique for interrogating the transcriptome on single cell level and for resolving the heterogeneity of various subsets of adaptive and innate immune cells involved in inflammatory networks in chronic inflammation (14). Consistently, while the first single cell being sequenced was reported in 2009 (15), more than 4000 Pubmed-indexed articles published in 2021 mention single-cell sequencing.

To aid in the interpretation of these big data, many tools have been written over the past years to support scRNA-seq analyses (16). Two programs are the backbone for most of these analyses: the Python library Scanpy (17) and the R package Seurat (18). To detect communities of cells with similar features, unsupervised clustering according to the Leiden or Louvain algorithms can easily be integrated into Scanpy- or Seurat-based pipelines. However, these approaches may sometimes be limited or time-consuming, when the goal is to analyze a specific subset of cells that does not directly match to the clusters identified.

We hypothesized that in these situations, manual selection of cells on any two-dimensional representation of single cell sequencing data might be a helpful additive tool for exploring the dataset. Although there are already some interactive single cell analysis tools, which are very user-friendly and allow to manually select cells by polygons without the need for much bioinformatics knowledge (19, 20), these applications are limited, when it comes to integrating other tools, changing analysis parameters or performing sophisticated downstream analyses. On the other hand, despite a huge variety of solutions for these advanced issues in Scanpy, there is currently no easy way to integrate manual cell selection by polygons in Scanpy-based pipelines. Collectively, there is an unmet need for a tool facilitating not only manual cell selection on single cell data, but also supporting downstream characterization and analyses of the selected population, e.g. in terms of (differential) gene expression, in Scanpy.

Thus, we aimed to close this gap and to simplify the analysis of immune cell subsets in scRNA-seq analyses in the context of chronic inflammation. To offer Scanpy users the ability to annotate their cells of interest by means of manual selection, we developed scSELpy (single cell SELection python). In addition to selecting cells of interest by drawing polygons around them, it supports further downstream analysis and the generation of publication-ready plots. Our data show that our tool is useful to analyze immune cell heterogeneity in IBD in scRNA-seq beyond conventional clustering and might therefore become an important application for future single cell transcriptomic analyses.

## Materials and methods

### scSELpy

scSELpy was developed as a Scanpy extension and solely uses libraries required by Scanpy such as matlibplot (21) and numpy (22) (**Table 1**). It allows the scSELpy user to select cells by drawing polygons on top of scatter plots or on either of the following dimension-reduced representations: UMAP (23), TSNE (24), PCA or other Scanpy-supported embeddings, limited to 2 dimensions. The selected cells will be annotated according to the names given to the drawn polygons that they are located within, separated by comma. Subsequently, this cell annotation will be stored as observations in the Anndata object (25). The coordinates of the polygon itself will be stored as unstructured data. Polygons are denominated as integers by default, converted to string. A scSELpy function allows the user to easily convert the default names saved in the annotations to custom names.

**Table 1:** Version list.

Name	Language	Version	scSELpy import
scselpy	Python	1.0.0	–
scanpy	Python	1.7.2	Yes
numpy	Python	1.21.1	Yes
matplotlib	Python	0.11.6	Yes
pandas	Python	1.2.4	No
scipy	Python	1.6.2	No
jupyter	Python	6.4.12	No
rpy2	Python	3.4.5	No
scirpy	Python	0.11.2	No
magic-impute	Python	3.0.0	No
sklearn	Python	1.0.2	No
scran	R	1.14.6	No

The invocation function of scSELpy mimics Scanpy’s plotting function to make it easy for the user to switch between using Scanpy and scSELpy. The scSELpy tool accepts all Scanpy parameters, except for the “Layer” parameter and offers additional parameters for fine tuning and re-plotting of polygons.

Upon invocation of scSELpy, it will determine if the user is running Python in a shell or in a notebook environment. For scSELpy to work on a notebook, it switches to an interactive matplotlib plotting backend. After the cell selection has been conducted, it will switch back to the default matplotlib plotting backend. On a Python shell such as ipython, a backend switch will not be conducted.

Afterwards, scSELpy will call Scanpy to create a plot of the specified embedding. While the plot is open, matplotlib’s function “ginput” is called, which will catch the coordinates of all mouseclicks on the plot. When the user is finished selecting the coordinates of a single polygon, the coordinates are sent to the plot function of matplotlib.pyplot, in order to draw the polygon on the Scanpy generated plot. After all polygons are drawn, scSELpy will switch back to a static backend if necessary. Subsequently it will call Scanpy and Matplotlib again to generate a final image for output.

For each polygon the contains_points function of Matplotlib is called, in order to determine which cells are located within which polygon. This function tests if a given cell coordinate of the passed embedding is located within the given polygon. The output are x boolean lists, where x is the amount of drawn polygons. These boolean lists are converted by scSELpy to a single list that contains, which cell is located in which polygon. The list is assigned to an observation in the anndata object, which is updated in place.

Additionally, scSELpy has a three functions for calculating the i) percentage of cells in each cluster or region, ii) percentage of cells expressing a given gene in each cluster or region, iii) transcripts per million (TPM) of a given gene in each cluster or region (**Table 2**). These functions can be used on any observation of the Anndata object and is therefore not exclusive to scSELpy generated regions.

**Table 2:** Supported read-outs for manual cell selections with scSELpy – example from **Figure 2B**.

	Percentage of cells	Percentage of cells expressing		TPM of	
		*CCR7*	*SELL*	*CCR7*	*SELL*
Selection	62.57	61.21	57.77	267.51	284.72
Other cells	37.43	19.38	25.19	61.81	107.08

### Data analysis

We analyzed two scRNA-seq datasets in this manuscript, which have been previously published under GSE162624 (26) and GSM6346300. The data were preprocessed and normalized in the same way as in the original study of GSE162624. The entire analysis was conducted on Jupyter notebook v6.4.12. Cells were selected and annotated using scSELpy. All plots were generated using Matplotlib, Scanpy and scSELpy. All data imputations were conducted by MAGIC (27). T cell receptor analyses were performed with Scirpy (28).

### Gene enrichment calculation

Assuming that one Unique Molecular Identifier (UMI) represents one detected mRNA transcript, the transcripts per million (TPM) for a given gene were calculated by dividing all UMIs of this gene in a specific population by all UMIs in the same population, multiplied by one million.


$$TPM=\left(\frac{\text{UMI count for given gene}}{\text{Total UMI count}}\right)*10^{6}$$

The enrichment for a given gene was calculated by dividing the TPM of a gene within a specific population by the TPM withing all cells outside that population.


$$Enrichment=\frac{\text{TPM of all cells in specific population}}{\text{TPM of all other cells outside of the population}}$$

### Gaussian mixture model

Cells were divided into groups using a gaussian mixture model with k-means as initializer based on the normalized mRNA-derived UMI count of two marker genes. This was done in Python with sklearn, using the GaussianMixture function from sklearn.mixture and the Kmeans function from sklearn.cluster.

### Availabilty of scSELpy

scSELpy is available for download at https://github.com/MarkDedden/scSELpy together with installation instructions, the data analysis pipeline and a link to the documentation, which includes a tutorial.

## Results

### Single cell selection in Python

To overcome the problem that standard scRNA-seq analysis pipelines, for example based on Scanpy, do not include tools to support manual cell selection from scatter or dimension reduction plots for further downstream analysis, we developed scSELpy as detailed in the Methods section.

### Positive selection with scSELpy

To explore and to demonstrate the functionality of scSELpy, we reanalyzed data (GSE162624) from a previous study (26), where CD3^+^CD4^+^CD45RO^+^α4^+^β7^+^ gut-homing memory T cells from the peripheral human blood were purified by fluorescence-activated cell sorting (FACS) and submitted to single cell transcriptomics.

**Figure 1:**
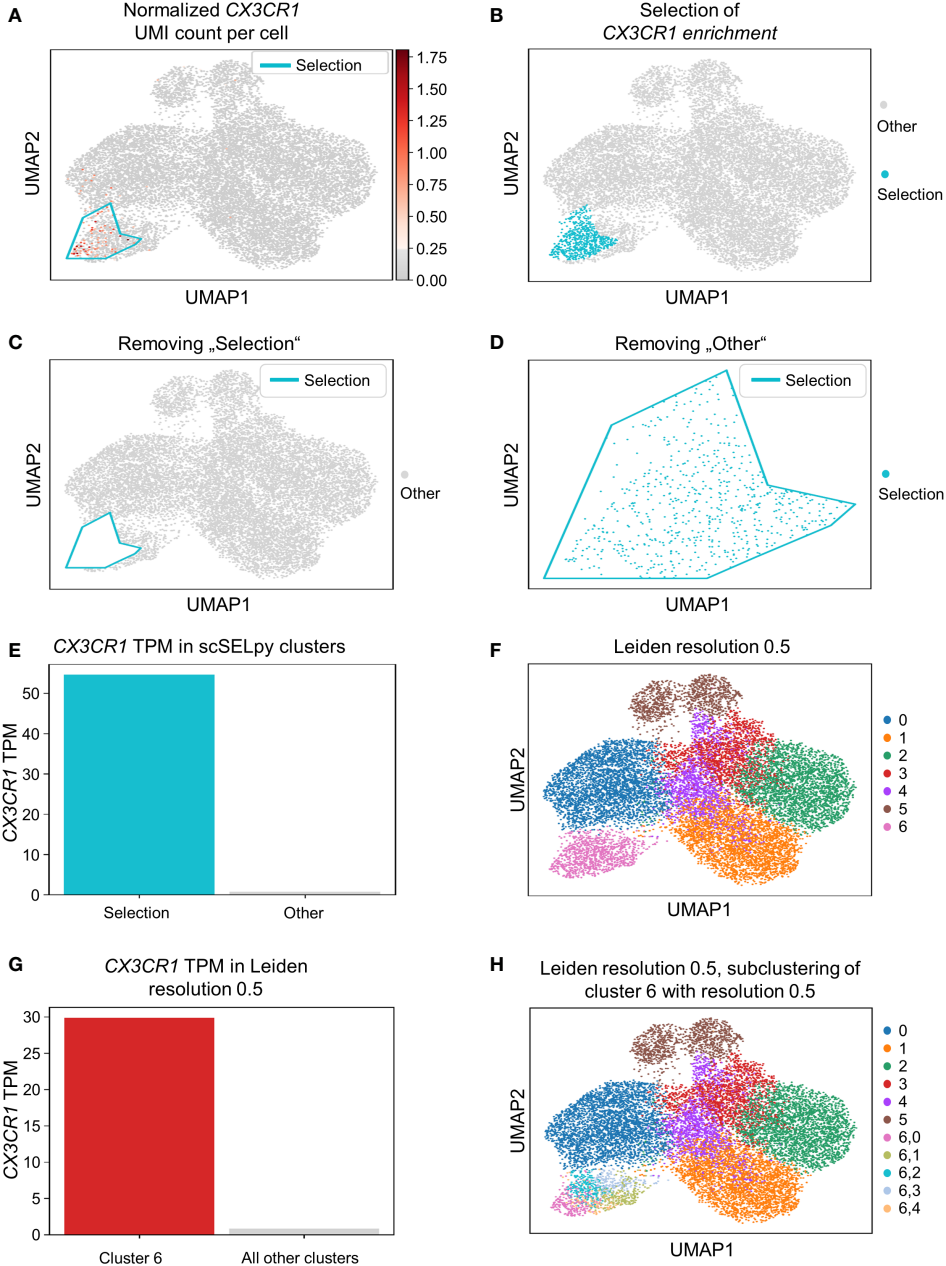
Positive selection of cell subsets on a Scanpy-generated UMAP plot with the scSELpy tool. **(A)** UMAP expression plot of *CX3CR1*. Regions with high *CX3CR1* expression were manually selected using scSELpy. **(B–D)** The cells located in the selected region can be highlighted **(B)**, removed **(C)**, or isolated **(D)** in downstream analysis. **(E)** Barplot with *CX3CR1* transcripts per million transcripts (TPM) for the selected cells versus all other cells. **(F)** Leiden clustering of the dataset with a resolution of 0.5. **(G)** Barplot with *CX3CR1* TPM for cluster 6 and all the other clusters together.**(H)** Subclustering of the leiden cluster 6 from **(E)**.

Based on a UMAP expression plot for *CX3CR1* generated by Scanpy, we selected a region high in cells expressing *CX3CR1* (**Figures 1A–D**). Indeed, a more than 90-fold increase in *CX3CR1* TPM were detected in the selected cells compared to the non-selected cells (**Figure 1E**, **Table 3**). Importantly, the region high in *CX3CR1* selected by the polygon gate was different from the clusters generated by the Leiden community detection algorithm with a resolution of 0.5 (**Figure 1F**) and the enrichment of *CX3CR1* transcripts in the manual selection compared with non-selected cells was higher than when comparing Leiden clusters with high vs. low *CX3CR1* expression (**Figure 1G**), where the enrichment was only below 40-fold increase in *CX3CR1* TPM. To investigate if further unsupervised subclustering would lead to an enrichment comparable to manual selection, we subclustered cluster 6 (**Figure 1H**) and calculated the enrichment for each subcluster. Even when combining the three subclusters (6,0, 6,2, 6,4) with the highest enrichment, we obtained only a 50.74-fold increase in *CX3CR1* TPM. Collectively, these data suggested that in specific scenarios, polygon gates drawn with scSELpy result in more specific positive selection of cell populations enriched for a certain gene than conventional clustering.

**Table 3:** Enrichment of *CX3CR1*.

Cluster	Enrichment [fold]
6	38.3
6,0	45.3
6,1	1.2
6,2	13.7
6,3	4.3
6,4	16.6
6,0 + 6,2 + 6,4	50.7
scSELpy selection	91.1

### Negative selection with scSELpy

**Figure 2:**
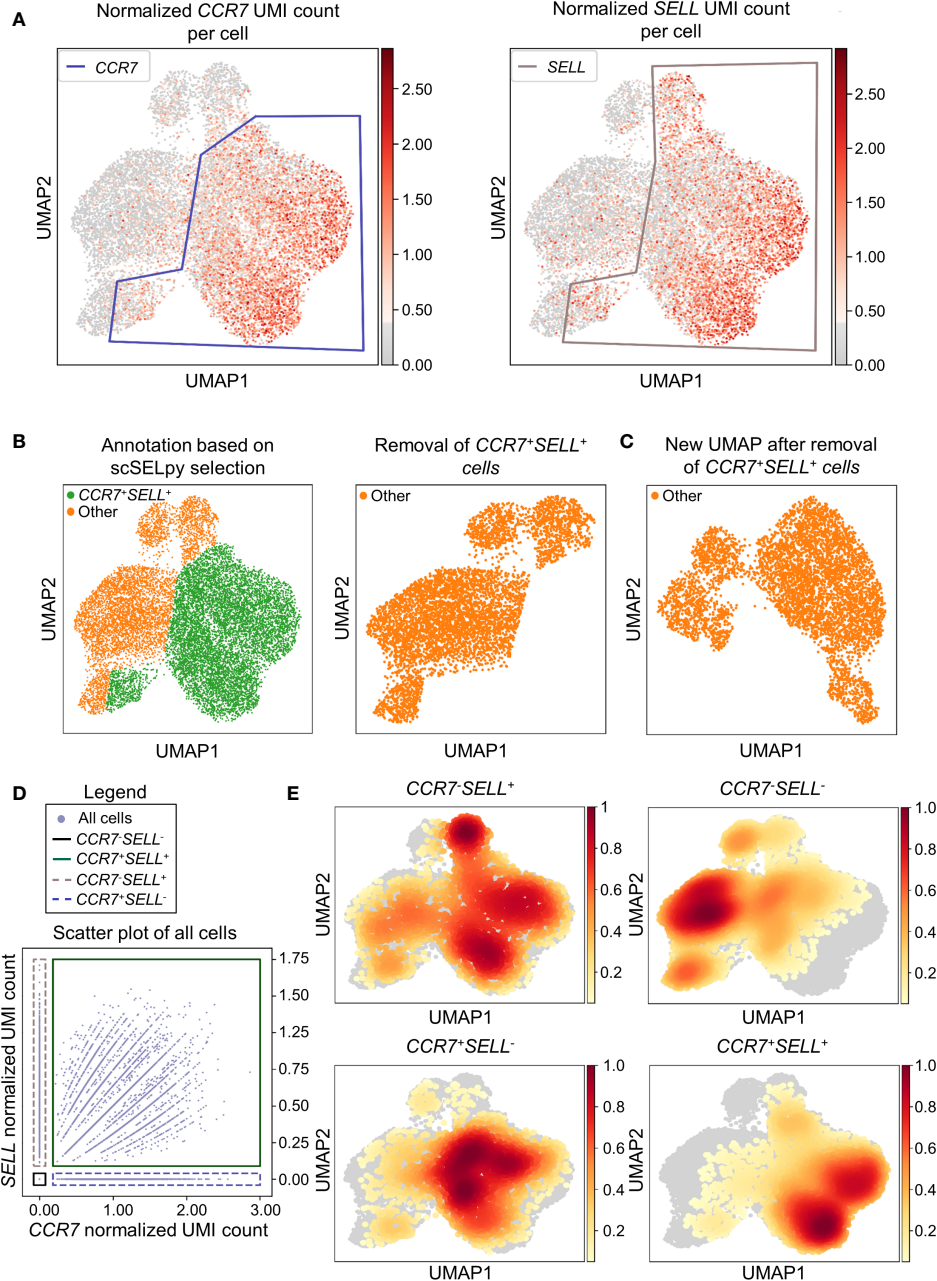
Selection and removal of central memory T cells from the dataset using scSELpy to obtain effector memory T cells. **(A)** Selection of regions enriched for cells expressing *CCR7* and *SELL* (CD62L, L-Selectin) on UMAP plots. **(B)** Identification and removal of the cells located in both the *CCR7* and *SELL* polygon as selected in A (green). **(C)** Re-analysis of the remaining cells from raw data. Normalization and UMAP dimension reduction is performed anew. **(D)** Scatter plot of all cells from the dataset with normalized *CCR7* and *SELL* expression on the x-axis and y-axis, respectively. On this scatter plot, scSELpy was employed to categorize cells with or without expression of *CCR7* and/or *SELL*. **(E)** UMAP density plots highlighting the cells belonging to the categories created in **(D)**. The parameter “vmin” to control the lower limit in the color scale was set to 0.05.

In a next step, we aimed to show that our application is also useful for the negative selection of cells of interest. Specifically, we sought to analyze T cell subsets relevant in the inflamed mucosa in IBD. Thus, since the dataset comprised peripheral blood memory T cells expressing the gut-homing marker α4β7, we aimed to identify T cells homing to the lamina propria by excluding central memory T (TCM) cells, which home to the gut-associated lymphoid tissue (29). To this end, we used polygon gates to mark regions high in expression of the TCM markers *CCR7* and *SELL* (CD62L) on UMAP plots (**Figure 2A**). The overlay of regions, where both genes were expressed in high levels was removed to obtain the cells equipped for access to the inflamed lamina propria (**Figure 2B** *)* as evident by a low prevalence of cells expressing CCR7 and/or SELL and low expression of these genes in the retained region *(* **Table 2**). The remaining cells were further re-analyzed from raw data, creating a new UMAP plot (**Figure 2C**).

To confirm that the selected region was also enriched for cells co-expressing *CCR7* and *SELL*, we employed scSELpy on scatter plots to “gate” for cells highly expressing *CCR7* and *SELL* (*CCR7* ^+^ *SELL* ^+^), highly expressing *CCR7* or *SELL* (*CCR7* ^+^ *SELL* ^-^, *CCR7* ^-^ *SELL* ^+^) and expressing *CCR7* and *SELL* at low levels or not at all (*CCR7* ^-^ *SELL* ^-^; **Figure 2D**). Subsequently, we depicted the presence of the cells from these four categories in density plots (**Figure 2E**). Here, the vast majority of *CCR7* ^+^ *SELL* ^+^ cells were located in the removed region, while most of the *CCR7* ^-^ *SELL* ^-^ cells plotted to the region that was kept, indicating that our tool had helped to correctly eliminate TCM cells.

As scRNA-seq suffers from drop-out events, where mRNA-transcripts that are present in a cell might not be detected, we employed data imputation using MAGIC (27) to verify that the selections made with scSELpy are not excluding cells falsely negative for *CCR7* and/or *SELL*. We overlayed the polygons of **Figure 2A** on the imputed data (**Figure S1A**) and further depicted the imputed data in a scatter plot and density plots (**Figures S1B, C**). Indeed, the enrichment of *CCR7* ^+^ *SELL* ^+^ in the overlap of the polygons was maintained, supporting the notion that manual selections predominantly include false negative cells (which is intended), but do not exclude them to a relevant degree.

### scSELpy allows for subphenotyping of T cells

**Figure 3:**
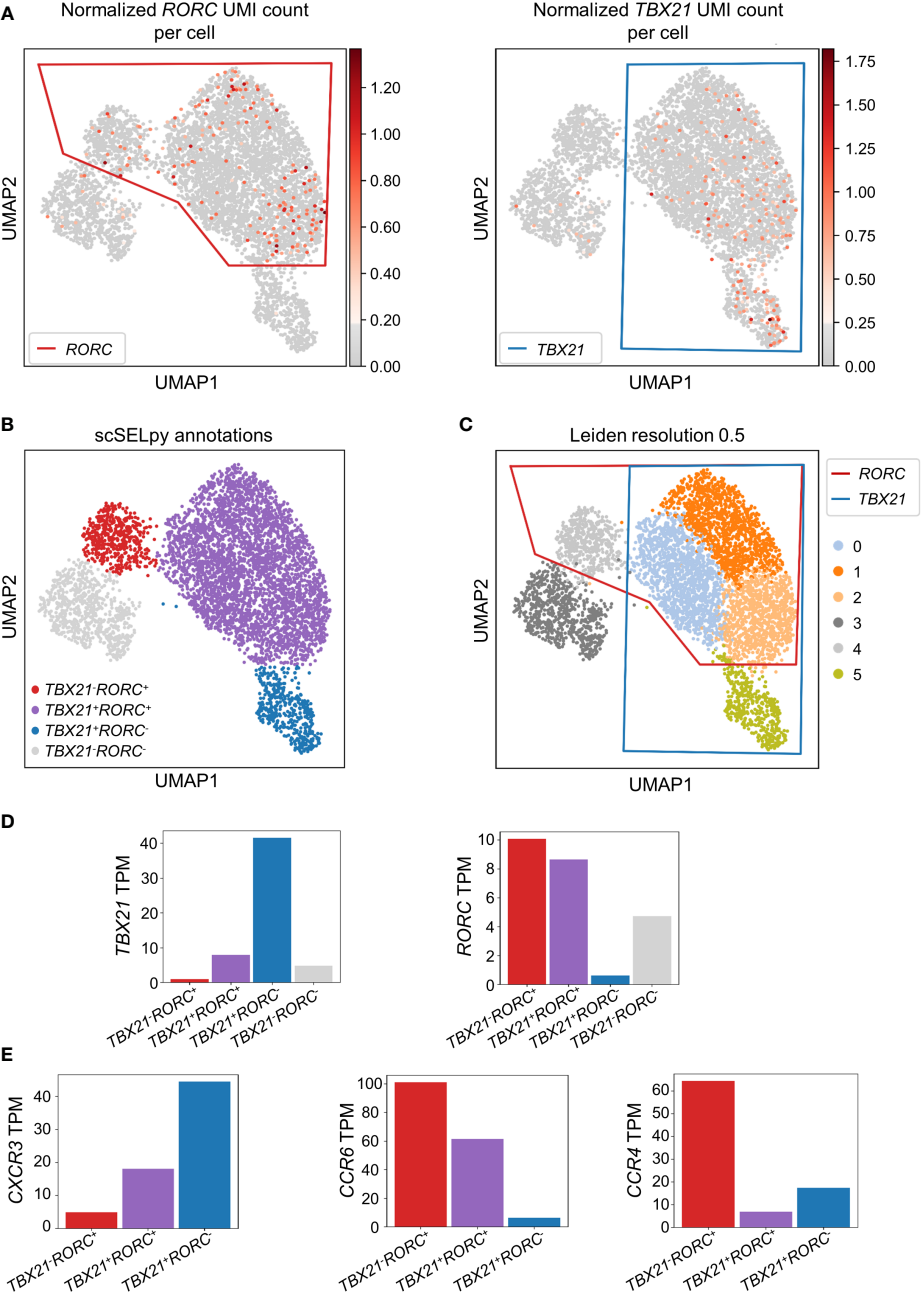
Identification and analysis of effector memory T cell subsets with scSELpy. **(A)** Selection of regions enriched in cells expressing the T cell transcription factors *TBX21* or *RORC* among effector memory T cells in the cells obtained in **Figure 2C**. **(B)** UMAP plot with cells annotated by scSELpy based on the selections made in **(A). (C)** Clustering of the cells based on the leiden algorithm with a resolution of 0.5. **(D)** Barplot showing the *TBX21* and *RORC* transcripts per million transcripts (TPM) for the populations identified in **(B)**. **(E)** *CXCR3*, *CCR6* and *CCR4* TPM in the indicated regions.

To confirm that the cells remaining after negative selection (**Figure 2C**) included tissue-homing TEM cells relevant in IBD and to characterize them, we explored the expression of *TBX21* and *RORC* as key transcription factors for TH1 and TH17 cells, respectively (30, 31). *TBX21* and *RORC* were expressed in overlapping regions that were manually selected by polygons (**Figure 3A**) resulting in the four populations of *TBX21^-^RORC* ^-^, *TBX21^+^RORC^-^*, *TBX21^-^RORC^+^* and *TBX21^+^RORC^+^* cells (**Figure 3B**). Interestingly, this matched well to Leiden clustering at a resolution of 0.5 (**Figure 3C**). Again, data imputation with MAGIC retrospectively supported the chosen manual selection (**Figure S1D**).

To validate enriched expression of *TBX21* and *RORC* in the selected populations, we calculated the TPM of the two genes. Indeed, mRNA expression of both transcription factors was substantially increased in the selected cell clusters (**Figure 3D**). This was consistent with the notion that *TBX21^+^RORC^-^*, *TBX21^-^RORC^+^* and *TBX21^+^RORC^+^* cells corresponded to TH1, TH17 and TH1/17 cells (a subset that has been described in the gut of patients with CD (32)), respectively. Thus, we further aimed to corroborate successful T helper cell subset identification by our tool and analyzed chemokine receptor expression in these three datasets. As expected based on previous reports (33), *TBX21^+^RORC^-^* cells were CXCR3^high^, but *CCR4^low^* and *CCR6^low^*, *TBX21^-^RORC^+^* cells *CCR6^high^* and *CCR4^high^*, but *CXCR3* ^low^ and *TBX21^+^RORC^+^* cells were *CXCR3^high^* and *CCR6^high^*, but *CCR4^low^* (**Figure 3E**).

Moreover, to further compare the capability of scSELpy to select cells enriched for a certain gene with another technique that is independent of spatially represented clusters or regions, we applied a Gaussian mixture model clustering with K-means as initializer for *TBX21* and *RORC* on the cells obtained in **Figure 2C**. However, in this case, the algorithm was not able to identify a specific regions enriched for *TBX21* and/or *RORC* (**Figure S2A**), strongly contradicting the impression supported by Leiden clustering and scSELpy selection that T helper cell populations cluster in defined regions. Thus, this scenario identified another situation, where scSELpy was better suited than established alternative solutions.

Collectively, these data showed that our tool had indeed identified TEM cells and was also able to further sub-cluster well-defined T helper cell subsets, which are relevant in IBD.

### scSELpy analysis confirms the phenotype of vedolizumab-resistant regulatory T cells

**Figure 4:**
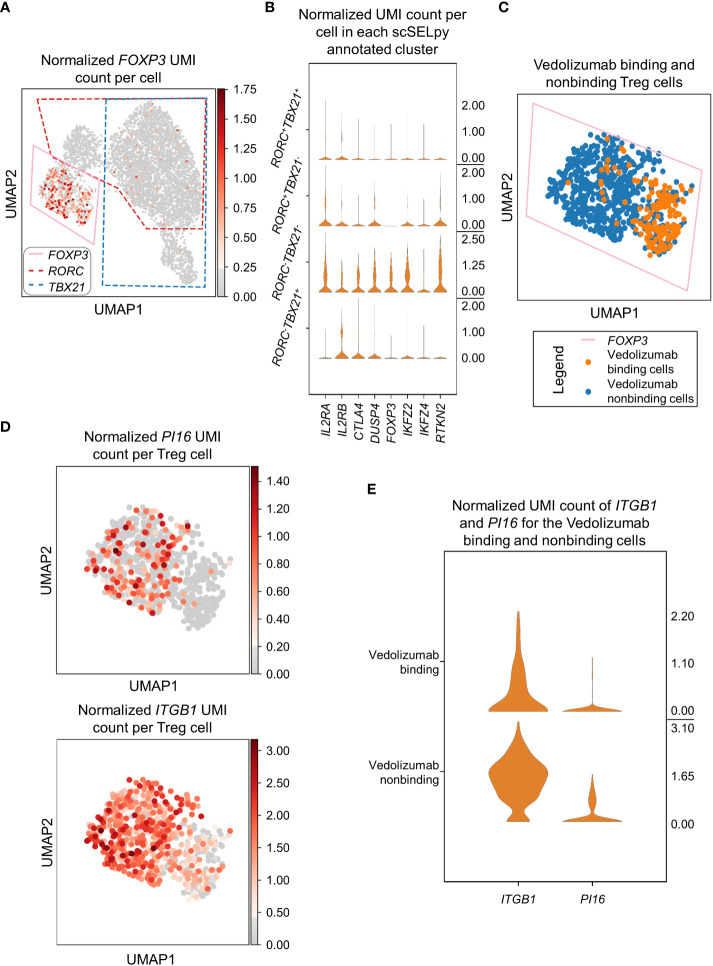
Selection and analysis of regulatory T cells with scSELpy. **(A)** Selection of the region enriched for *FOXP3* expression, using the UMAP representation presented in **Figure 2C**. The selections for *RORC* and *TBX21* from **Figure 3A** are marked with dotted lines. **(B)** Violin plots of the expression of eight genes associated with Tregs in the four regions determined based on *TBX21* and *RORC* expression. **(C)** Depiction of cells binding vedolizumab or not as determined by FACS sorting prior to sequencing on the population selected in **(A)**. **(D)** Expression of *ITGB1* and *PI16* in the cell population selected in **(A)**. **(E)** Stacked violin plot of *PI16* and *ITGB1* expression for Vedolizumab-binding cells and cells that do not bind Vedolizumab.

In a next step, we wanted to find out, what kind of cells were represented in the *TBX21^-^RORC^-^* cluster. Since, regulatory T (Treg) cells are another important gut-homing T cell population and play an important role in the resolution of intestinal inflammation (8, 10), we explored the expression of the key Treg transcription factor *FOXP3*. Not very surprising, we detected markedly enriched *FOXP3* expression and manually selected the subset for downstream analysis (**Figure 4A**). In further support of the Treg nature of these cells, several other Treg-associated molecules were expressed to significantly higher levels than in the other T cell subsets (**Figure 4B**).

Since the dataset consisted of sorted cells binding or not binding the anti-α4β7 integrin antibody vedolizumab, which is used for clinical therapy of IBD (34, 35), and we had previously shown in that dataset that a subset of Tregs is enriched for vedolizumab-resistant cells (26), we now explored whether scSELpy-based analysis comes to the same conclusion. Thus, we depicted vedolizumab binding cells and vedolizumab non-binding cells in our Treg population. Consistent with our previous analysis, a majority of Tregs did not bind vedolizumab (**Figure 4C**). Moreover, *ITGB1* and *PI16*, two genes that had been found to mark those vedolizumab-resistant Tregs were clearly enriched in the Treg fraction that did not bind vedolizumab (**Figure 4D, E**). Taken together, these data showed that scSELpy is able to reproduce earlier findings and is, thus, a valid tool for advanced analyses of single cell transcriptomics in general and in the context of IBD in particular.

### scSELpy helps analyzing protein expression and TCR sequencing data

**Figure 5:**
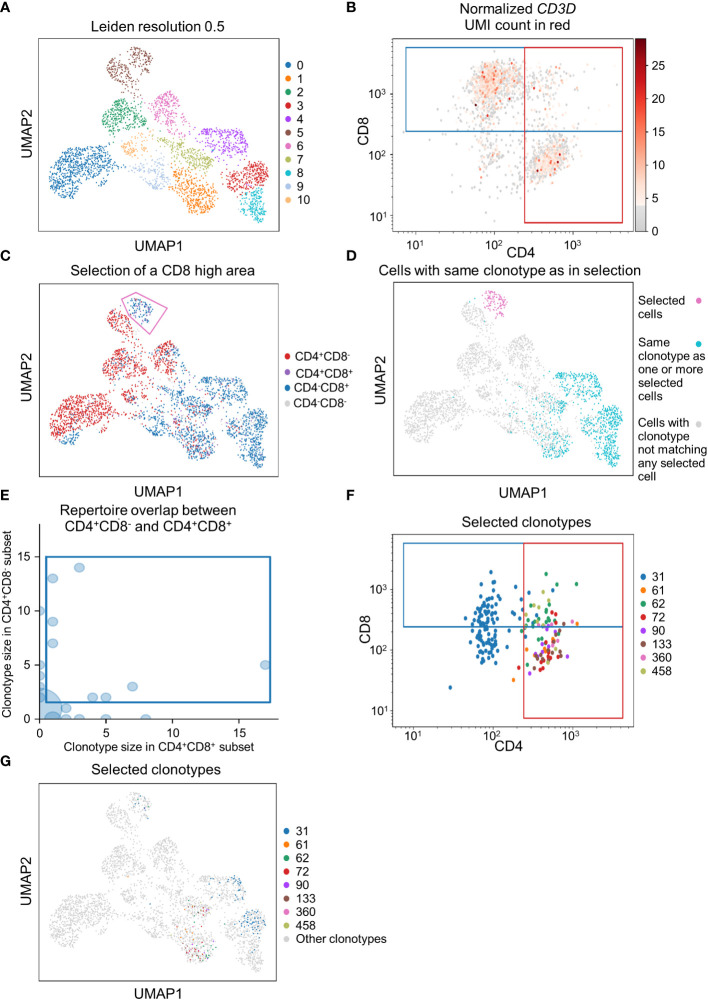
Applying scSELpy on T cell receptor (TCR) sequencing and antibody capture data. The single cell RNA sequencing data of PBMCs in blood from patients treated with Immune checkpoint inhibitors was retrieved from GSM6346300. Only cells, for which a TCR is detected are used in these plots. **(A)** UMAP plot of the cells; clusters are defined by leiden with a resolution of 0.5. **(B)** Scatter plot with the detected CD4 and CD8 antibody UMIs on the x-axis and y-axis, respectively. Gates set with scSELpy split the data up into CD4^+^CD8^+^ double positive cells, single positive CD4^+^CD8^-^ cells, single positive CD4^-^CD8^+^ cells and CD4^-^CD8^-^ double negative cells. The *CD3D* mRNA derived UMI count is highlighted in red. **(C)** Visualization of the four groups defined in **(A)** on the UMAP plot. A group of cells is selected with scSELpy. **(D)** UMAP representation of T cells selected in **(C)** (magenta) together with all other T cells also expressing one of the TCRs present in the selection (blue). Cells colored in grey do not have a clonotype that matches a T cell in the selected region. **(E)** TCR repertoire of CD4^+^CD8^-^ cells plotted against TCR repertoire of CD4^+^CD8^+^ cells for each clone ID using Scirpy. Clone IDs that are present in both cell populations were selected using scSELpy. **(F)** Mapping of the cells selected in **(E)** on the scatter plot of CD4 and CD8 antibody UMIs with the gates kept as in **(B)**. **(G)** UMAP representation of the T cells selected in **(E)**.

To demonstrate that scSELpy is able to analyze protein expression identified by antibody-oligo capture, we analyzed the dataset GSM6346300 with scSELpy (36). In that dataset, PBMCs from four patients were isolated from the blood and sequenced on a 10x Chromium controller. This included V(D)J single-cell T cell receptor (TCR) sequencing and Feature Barcoding to capture the protein expression of CD4, CD8 and CD45RA. We loaded the TCR data using Scirpy, merged it with the scRNA-seq dataset and removed all cells that had no TCR detected using the “has_ir” observation created by Scirpy. We proceeded under the assumption that the remaining subset consists of only T cells. We used Leiden clustering with a resolution of 0.5 to assign clusters on a two dimensional UMAP plot (**Figure 5A**).

Using scSELpy, we drew a gate for CD8^+^ cells and a gate for CD4^+^ cells on a scatter plot of raw CD8 and CD4 antibody interaction-derived UMI counts with a logaritmic axis (**Figure 5B**). We highlighted the *CD3D* (**Figure 5B**), *CD8A*, *CD8B* and *CD4* mRNA-dervied UMI count (**Figure S2B**) to show that the mRNA expression of *CD8* and *CD4* is enriched in their respective gates. The CD4^+^CD8^-^, CD4^-^CD8^+^, CD4^+^CD8^+^ and CD4^-^CD8^-^ populations were now mapped back to the initial UMAP plot (**Figure 5C**, **Figure S2C**). Interestingly, in this case, these populations were well aligned with those identified by the gaussian mixture model clustering with K-means as initializer (**Figure S2D**). To demonstrate that this is a feasible starting point for further downstream analyses and that scSELpy can also assist in understanding TCR sequencing data, we manually selected a region of cluster 5 enriched for CD8^+^ T cells with scSELpy (**Figure 5C**). Building on the Scanpy-based library for T cell receptor-sequencing data analysis, Scirpy, we now plotted all other cells sharing a clonotype with any of the cells in this area and found that these cells mainly map to the clusters 1, 3, 4 and 8 (**Figure 5D**).

To further demonstrate the potential to use scSELpy for analyzing combined TCR and scRNA sequencing data, we used Scirpy’s repertoire_overlap function to plot the T cell repertoire overlap between the CD4^+^CD8^-^ gate and the CD4^+^CD8^+^ cells. Using scSELpy on this plot, we selected clonotypes that are present in cells of both gates (**Figure 5E**) and plotted them in the CD8 versus CD4 scatterplot (**Figure 5F**), keeping the gates as set in **Figure 5B**, and in the UMAP plot (**Figure 5G**) as potential starting points for further selection and analysis procedures.

Collectively, these approaches demonstrated that scSELpy can be used as a convenient and flexible tool helping in the exploration and analysis of multi-dimensional single cell sequencing data.

## Discussion

Single cell RNA sequencing has revolutionized immunological research and has helped to substantially increase the resolution of immune cell phenotyping (14). However, this comes at the cost of complex *in silico* analyses to be performed.

Here, we introduce a new tool called scSELpy designed to enable the manual selection of cells analyzed by single cell transcriptomics in scatter plots or dimension reduction representations to allow further downstream analysis. As such, it is inspired by the “gating” used in multicolor flow cytometry as the most widely used technique to interrogate protein expression on single cell level (37). In flow cytometry, such gating serves to select certain cell populations in a hierarchical manner and to determine the abundance and phenotype of cell subsets in this way (38).

Manual cell selection in scatter plots visualizing the expression of two different genes per cell as offered by scSELpy comes most closely to this function. However, the high number of parameters analyzed by single cell RNA sequencing also imposes the necessity to include dimension reduction techniques to appropriately visualize and analyze relationships between the single cells (23, 24). Consistently, scSELpy also offers manual cell selection on dimension-reduced UMAP or t-SNE plots. Yet, it needs to be considered that in this case “gating” will not result in the binary selection of cells with and without expression of one or more genes (or high or low expression), but only in the selection of a population enriched in cells expressing those genes. This population will also include closely related cells, in which expression of the gene has not been detected, which might be due to absent expression or expression below the detection threshold of single cell RNA sequencing (39). Our findings with imputed data further support this notion, since the polygons drawn before data imputation captured the majority of false-negative cells and missed only few of them. Taken together, these aspects emphasize that, while similar in handling, “gating” on scRNA-seq data, is clearly different from flow cytometry gating.

Importantly, scSELpy also supports the analysis of single cell data including TCR sequencing or sequencing of surface proteins detected by antibody-oligo reaction. Thus, in the specific case of protein expression analysis, scSELpy can actually be used for binary gating of surface expression markers very similar to flow cytometry. With regard to TCR analyses, we show that scSELpy can identify and plot clonotypes in various representations and might thus help to explore their role and function. Again, this is not an exclusive feature of scSELpy, since for instance clonotype overlap in different cell types can also be identified using Scirpy’s clonotype_imbalance function. Thus, it is important to understand scSELpy as a complementary tool that can be used together with other important approaches to reach a deeper understanding of the data.

Several publicly available applications such as the Loup Browser offered by 10x Genomics, CELLxGENE, the UCSC Cell Browser (19, 20) or Shiny-based applications for scRNA-seq such as SCHNAPPs (40) are graphical user interface platforms for single cell analysis, of which the first three mentioned offer similar tools for manual cell selection. However, while those applications are easy to handle also for researchers without training in bioinformatics, a limitation of them is that further downstream analyses are not supported. Thus, scSELpy has been designed for use on the Scanpy platform, one of the standard applications used for the analysis of single cell RNA-seq data (17) and integrates a workflow to enable easy downstream phenotyping of selected cells such as further sub-clustering, re-plotting or differential expression analysis.

A standard technique to identify specific cell populations on single cell transcriptomic data is clustering according to the Leiden or Louvain algorithm (41). Depending on the resolution used, these algorithms dissect the overall cell population into several clusters, which can subsequently be extracted and further analyzed. It is important to mention that scSELpy is not at all meant to replace such clustering-based identification and selection of cell populations, but as an additional tool that might be helpful in certain situations.

One key difference to scSELpy is that Leiden or Louvain clustering are unsupervised and thus unbiased. In consequence, one might mention that scSELpy unnecessarily introduces bias into single cell RNA sequencing analysis by allowing to select cells based on one or more deliberately chosen genes. While this has to been accepted as a potential limitation and to be kept in mind during analysis, it is also important to note that conventional clustering might not always perfectly capture biological processes that are not dominating the phenotype of cell subsets or are shared between subsets. For example, this might be processes of cell migration such as gut homing. Consistently, our data show that manual cell selection by scSELpy helps to increase the enrichment of specific gene expression in the selected populations. This might be of particular value in a time, where the re-analysis of previously published datasets from a novel perspective is becoming more and more important (42).

It is essential to underscore that in many situations (e.g. as documented in **Figure 3C** or **Figure S2D**) there exist conventional alternatives such as Leiden or Louvain clustering or k means clustering that lead to similar results as manual cell selection with scSELpy. Thus, in these situations, the benefits (fast, easy) and the limitations (subjectivity) associated with the use of scSELpy must be carefully weighed. However, as we show (**Figure 1E-H**, **Figure S2A**), there are also scenarios, where scSELpy results in superior selection of enriched populations. Similarly, one can also consider situations, where the use of scSELpy is limited by very rare expression of a gene or equal distribution over the dimensionality-reduced space and alternative ways of cell selection will be more helpful. In consequence, we think that scSELpy is a valuable part of the toolbox in state-of-the-art single cell analysis that should especially be used in situations, where conventional community detection or cell type identification are not possible, sub-optimal or very time-consuming or where the role of a gene regardless of the association to a specific (sub-)community is explored.

We demonstrate the feasibility of our approach in a dataset characterizing gut-homing memory T cells from the peripheral blood, a cell population that is of particular interest for the pathogenesis of IBD and has become a therapeutic target by blocking its gut homing through the anti-α4β7 integrin antibody vedolizumab (7). In addition to proving the suitability of scSELpy for appropriate positive and negative selection strategies, we also show that our tool is helpful in supporting the analysis of cell populations such as TH1, TH17 or TH1/17 cells, all of which have been demonstrated to crucially implicated in IBD (30–32). Thus, scSELpy might help to obtain further insights into immune cell regulation in IBD and other chronic inflammatory diseases in the future. Moreover, in regulatory T cells our tool was able reproduce the earlier finding that α4β7-expressing regulatory T cells are enriched in cells “resistant” to vedolizumab and that β1 integrin and PI16 are highly expressed in those cells (26). It might therefore also be employed for future translational studies in the field of IBD aiming at dissecting the mechanisms of state-of-the art treatment options at higher resolution.

Taken together, to the best of our knowledge, scSELpy is the first tool that can offer Scanpy-based manual cell selection. Based on the data presented, we project that, when used intentionally, it might broadly support and facilitate single cell transcriptomic analyses for many researchers in the field of immunology in general and in IBD in special.

## Data availability statement

The datasets presented in this study can be found in online repositories. The names of the repository/repositories and accession number(s) can be found in the article/**Supplementary Material**.

## Author contributions

MD developed scSELpy and performed the analyses. MD and SZ designed the analyses and interpreted the data together with MW, TM and MN. MD and SZ drafted the manuscript; All authors contributed to the article and approved the submitted version.

## Supplementary material

Supplementary Figure 1 Visualization of how the previously set selections in Figures 2, 3 and look with imputed data. **(A)** The same UMAP plot and selection as in Figure 2D, where now the imputed data is visualized. **(B)** Same scatter plot as in Figure 2D, where now the imputed data is used. **(C)** UMAP density plots highlighting the cells belonging to the categories created in **(B)**. The parameter “vmin” to control the lower limit in the color scale was set to 0.05. **(D)** The same UMAP plot and selection as in Figure 3A visualizing the imputed data. The parameter “vmax” to control the upper limit in the color scale was set to 0.12.

Supplementary Figure 2
**(A)** Guassian mixture model of *RORC* and *TBX21* mRNA-derived UMI count with the data from Figure 3, plotted on the same UMAP plot. **(B)** The same plot as Figure 5A, with the modification that the *CD8A* (left), *CD8*B (middle) and *CD4* (right) mRNA derived UMI count is highlighted as indicated. **(C)** UMAP plot with CD8 (right) and CD4 (left) antibody UMI counts highlighted in red. **(D)** Guassian mixture model of *CD4* and *CD8A* mRNA-derived UMI count with the data from Figure 5B, plotted on the same UMAP plot.
