# Supplementary figures and images for: Manual cell selection in single cell transcriptomics using scSELpy supports the analysis of immune cell subsets
Source: Front Immunol. 2023 Apr 25;14:1027346. doi: 10.3389/fimmu.2023.1027346 (PMC10166880; doi:10.3389/fimmu.2023.1027346)

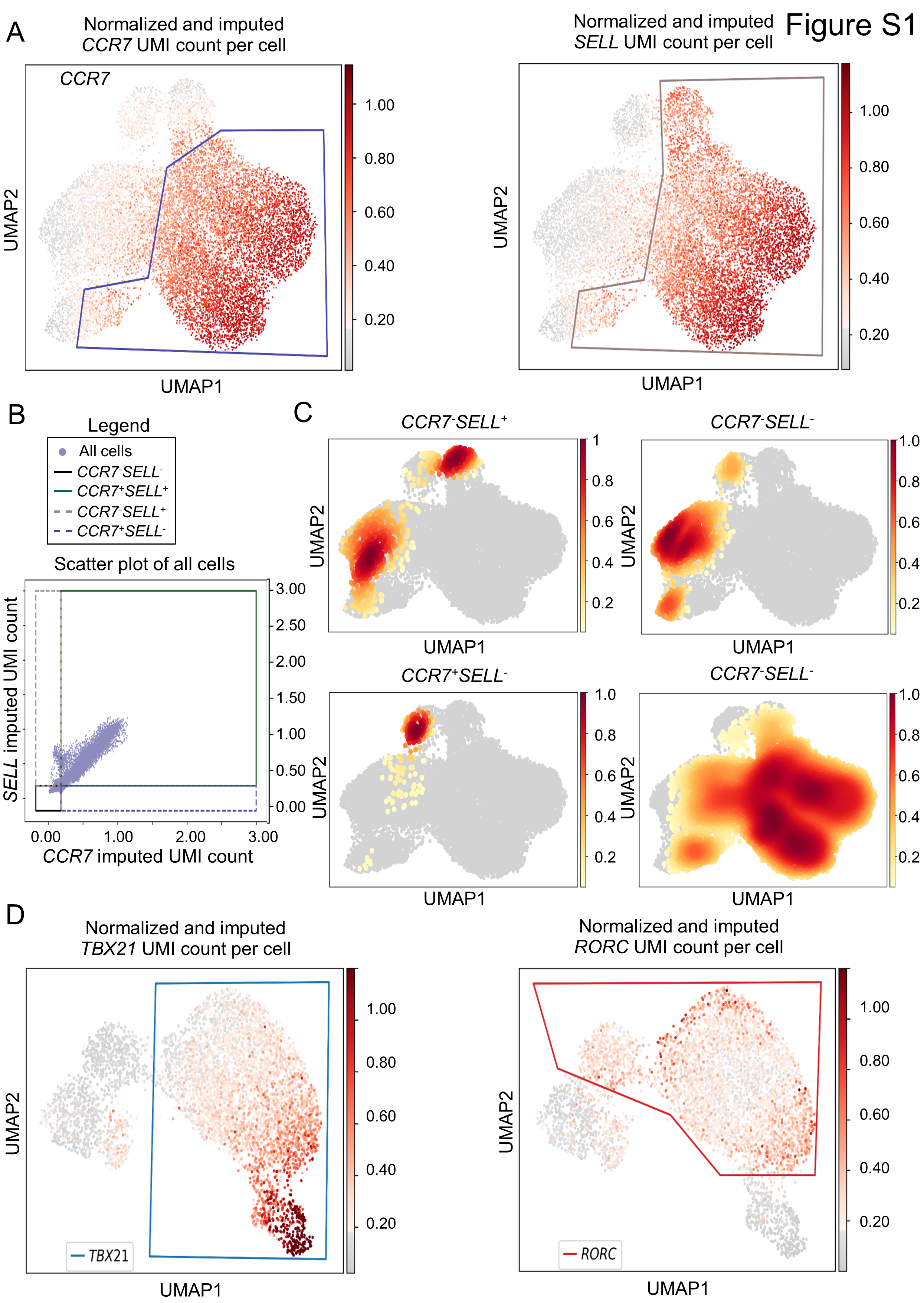

Supplement: Supplementary Figure 1 — Visualization of how the previously set selections in Figures 2, 3 and look with imputed data. (A) The same UMAP plot and selection as in Figure 2D, where now the imputed data is visualized. (B) Same scatter plot as in Figure 2D, where now the imputed data is used. (C) UMAP density plots highlighting the cells belonging to the categories created in (B). The parameter “vmin” to control the lower limit in the color scale was set to 0.05. (D) The same UMAP plot and selection as in Figure 3A visualizing the imputed data. The parameter “vmax” to control the upper limit in the color scale was set to 0.12. [file Image_1.tif]

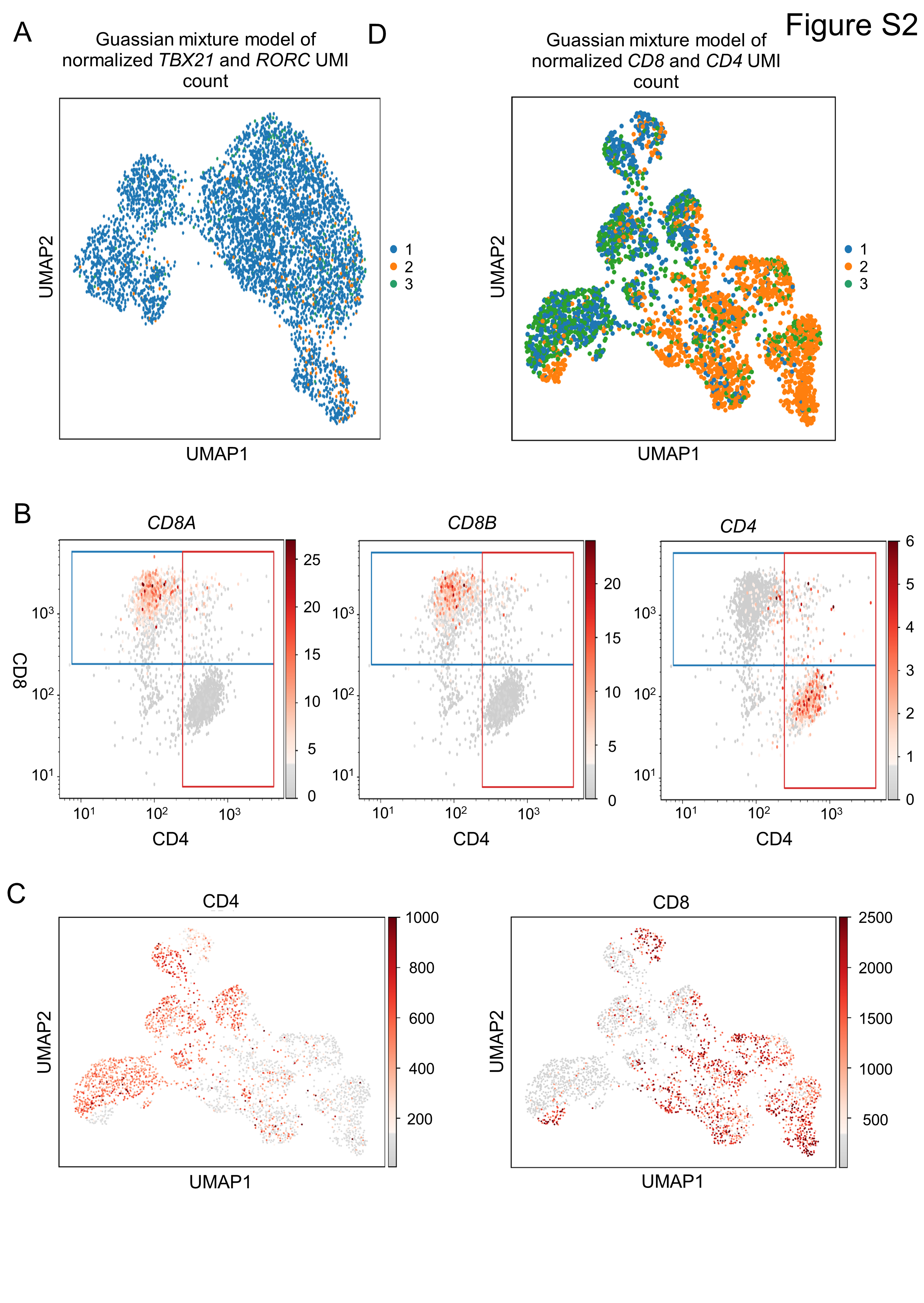

Supplement: Supplementary Figure 2 — (A) Guassian mixture model of RORC and TBX21 mRNA-derived UMI count with the data from Figure 3, plotted on the same UMAP plot. (B) The same plot as Figure 5A, with the modification that the CD8A (left), CD8B (middle) and CD4 (right) mRNA derived UMI count is highlighted as indicated. (C) UMAP plot with CD8 (right) and CD4 (left) antibody UMI counts highlighted in red. (D) Guassian mixture model of CD4 and CD8A mRNA-derived UMI count with the data from Figure 5B, plotted on the same UMAP plot. [file Image_2.tif]
